# *Helicobacter pylori* Promoted miR-196a/b-5p Expression and Accelerated Tumorigenesis of the Gastric Mucosa by Targeting IGF2BP1 and Activating PI3K-Akt Signaling Pathway

**DOI:** 10.5152/tjg.2025.24397

**Published:** 2025-05-05

**Authors:** Ke Chen, Jia-Wei Chen, Yao Shen, Yun-Fei Wang, Sheng-Rong Dong, Xiao-Xue Zhang, Chen-Yang Li, Xiao-Juan Gao, Jia-Min Zhao, Yu-Nan Zhang, Wen-Ying Tian, Jia-Le Lv, Qiang Zhan, Fang-Mei An

**Affiliations:** 1 Department of Gastroenterology, Affiliated Wuxi People’s Hospital of Nanjing Medical University, Wuxi People’s Hospital, Wuxi Medical Center, Nanjing Medical University, Jiangsu, China; 2 Jiangsu Branch of the National Clinical Research Center for Digestive Disease, Jiangsu, China; 3 Department of Gastroenterology, The First Affiliated Hospital of Nanjing Medical University, Jiangsu, China

**Keywords:** b-5p, GC, *H. pylori*, IGF2BP1, precancerous

## Abstract

**Background/Aims:**

The study focuses on examining the impact of *Helicobacter pylori* (*H. pylori*) on the modulation of the miRNA expression profiles while also unraveling the associated pathways that play a significant role in initiating and driving the development of gastric cancer (GC).

**Materials and Methods:**

An in-depth analysis of miRNA expression profiles in gastric tissue samples from patients with chronic superficial gastritis (CSG), chronic atrophic gastritis (CAG), dysplasia (Dys), or GC was conducted. The carbon-13 urea breath test was used to identify* H. pylori* infection, and the participant cohort was characterized by the presence of *H. pylori* infection. Additionally, the role of miR-196a/b-5p in GC carcinogenesis was investigated.

**Results:**

A total of five miRNAs—miR-196a-5p, 196b-5p, 224-5p, 424-3p, and 941—demonstrated marked elevation in CSG, CAG, Dys, and GC. miR-196a/b-5p was observed to be upregulated in GC cells following *H. pylori* infection, as well as in Dys and GC tissue samples from patients harboring *H. pylori*. miR-196a/b-5p can expedite GC progression. Insulin-like growth factor 2 mRNA-binding protein 1 (*IGF2BP1*), the target gene of miR-196a/b-5p, diminishes the proliferation capability of GC cells; however, miR-196a/b-5p can partially counteract this effect. miR-196a/b-5p activates the PI3K-Akt pathway, while IGF2BP1 inhibits the expression of these proteins.

**Conclusion:**

The levels of miR-196a/b-5p were observed to escalate following *H. pylori* infection, subsequently fostering the progression of GC by specifically targeting IGF2BP1 and triggering the PI3K-Akt signaling cascade.

**Main Points**

- The connection between *H. pylori* and miRNA during the carcinogenesis of gastric mucosa was investigated.
- The regulatory role of miR-196a/b-5p/IGF2BP1 in the development and advancement of gastric cancer was explored.

## Introduction

Gastric cancer (GC) holds the position of being the third most common cause of cancer-related mortality worldwide.^1^ In China, both the occurrence and fatality rates are high, therefore making prompt diagnosis and treatment of GC crucial.^2^ miRNAs can influence the oncogenesis, development, diagnosis, treatment, and prognosis of GC. For instance, miR-761 positively affects the proliferation of GC cells by negatively regulating glycogen synthase kinase 3β. Additionally, miR-21-5p directly targets and inhibits pyruvate dehydrogenase A1 and SMAD7, thereby enhancing glycolysis and promoting cell proliferation in GC. Furthermore, miR-23, miR-27a, and miR-24 facilitate GC cell proliferation by inhibiting the suppressor of cytokine-induced signaling 6.^3^ However, only few reports on the expression of miRNAs in precancerous lesions of the gastric mucosa are available.

Helicobacter pylori(*H. pylori*) is a Gram-negative bacterium that resides in the human gastric mucosa. A systematic review and meta-analysis indicated that the overall prevalence of* H. pylori* infection across 62 countries is 48.5%.^4^ The World Health Organization classifies it as a group 1 carcinogen concerning GC.^5^ A study evaluated the trends in global prevalence of* H. pylori* infection among adults and children/adolescents and examined its relationship with GC incidence. The findings revealed that the prevalence of *H. pylori* in adults has decreased from 52.6% before 1990 to 43.9% between 2015 and 2022, while remaining unchanged in children and adolescents. Concurrently, the incidence of GC has declined globally, and in several countries prevalence of *H. pylori* has also decreased.^6^ Another investigation demonstrated that *H. pylori* infection can lead to differential expression of miRNAs between GC tissue and the non-cancerous gastric mucosa.^7^ *Helicobacter pylori* infection influences miRNA expression in gastric mucosal cells through various epigenetic, transcriptional, and post-transcriptional mechanisms, through interactions with virulence factors and inflammatory mediators or through other mechanisms. However, a comprehensive understanding of the regulatory mechanisms by which *H. pylori* affects miRNAs is lacking.^8^

In this research, a thorough investigation was conducted to pinpoint the critical miRNAs found within the gastric precancerous and cancerous tissues. Furthermore, the contributory function of* H. pylori* in miRNA expression was explored. The role of the *H. pylori*/miRNA regulator axis in the tumorigenesis of GC was aimed to be explored.

## Materials and Methods

### Ethical Approval

This study was approved by the Ethics Committee of the affiliated Wuxi People’s Hospital of Nanjing medical university (date: August, 08, 2018; number: KY23106). Prior to commencing the experimental procedures, each participant involved in the research provided their formal agreement by signing an informed consent document.

### Clinical Specimens

All patients included in the study underwent gastroscopy due to symptoms of upper gastrointestinal discomfort such as vomiting, belching, abdominal pain, and bloating and/or due to clinical symptoms such as fatigue and anemia, without any signs of gastrointestinal bleeding such as hematemesis or melena. Tissue specimens of gastric mucosa were collected via endoscopic biopsy in the Digestive Endoscopy Center from January to December 2019 and January to October 2022.

For the miRNA microarray analysis, biopsies were obtained from the patients after an endoscopic biopsy at Wuxi People’s Hospital between October–December 2019 and September–October 2022. The samples were divided into 4 groups according to the pathological diagnosis: chronic superficial gastritis (CSG), chronic atrophic gastritis (CAG), dysplasia (Dys) and GC. All specimens originated from patients confirmed to be *H. pylori*-positive. For each category, 6 specimens were collected and subsequently preserved in RNAlater (AM7020, Thermo Fisher Scientific, USA) for sequencing. The sequencing data is accessed through number GSE164166.

A total of 85 CSG specimens *(H. pylori* positive = 35, *H. pylori* negative = 50), 26 CAG specimens (*H. pylori* positive = 17, *H. pylori* negative = 9), 32 Dys specimens (*H. pylori* positive = 14, *H. pylori* negative = 18), and 27 GC and matched adjacent gastric tissues (*H. pylori* positive = 16, *H. pylori* negative = 11) were obtained from the patients by endoscopic biopsy at the XXX between January to December 2019 and September to October 2022. The tissue specimens were stored in RNAlater at −80°C for the miR-196a/b-5p expression RT-qPCR verification.

A total of 15 matched Dys and adjacent normal tissues, as well as the 16 GC and matched adjacent normal tissues described above, were obtained from *H.* pylori-positive patients by endoscopic biopsy at the Wuxi People’s Hospital between January to December 2019 and September to October 2022. The tissue specimens were stored in RNAlater at −80°C for insulin-like growth factor 2 mRNA-binding protein 1 (IGF2BP1) mRNA RT-qPCR detection.

### Total RNA Extraction from Tissues, miRNA Sequencing, and Real-Time Quantitative Polymerase Chain Reaction

Total RNA from gastric tissue or cells was extracted using Trizol regiment (Invitrogen, Paisley, UK) according to the manufacturer’s instructions.

Six tissue samples were selected from each group of CSG, CAG, Dys, and GC for miRNA microarray analysis (Guangzhou Ribobio Co., Ltd., Guangdong Province, China). Small RNA libraries were generated with the aid of the NEBNext Multiplex Small RNA Library Prep Set for Illumina, supplied by New England Biolabs in the United States. The resultant libraries were then sequenced on an Illumina HiSeq 2500 system.

The synthesis of single-stranded complementary DNA (cDNA) was carried out using the HiScript II Q Select RT SuperMix for qPCR (with gDNA wiper) kit (R233-01, Vazyme). Real-time quantitative polymerase chain reaction (qPCR) was conducted according to the manufacturer’s instructions using the ChamQ Universal SYBR qPCR Master Mix kit (Q711-02, Vazyme). The U6 gene functioned as the reference gene for normalization of miR-196a/b levels. Simultaneously, β-actin was employed as the normalizing control when assessing IGF2BP1 expression (F-GCGGCCAGTTCTTGGTCAA, R-TTGGGC ACCGAATGTTCAATC). All RT-qPCR assays were performed in triplicate.

### Bioinformatics Analysis

This research utilized the Kyoto Encyclopedia of Genes and Genomes (KEGG) biological pathway database (http://www.genome.jp/) for the analysis of biological pathways. The Gene Ontology analysis was employed to perform functional annotation for each gene. The genes targeted by miR-196a/b-5p were identified using 4 publicly available databases: miRBD (http://mirdb.org/), miRWalk (http://mirwalk.umm. uni-heidelberg.de/), TargetScan (http://www.targetscan.org/vert_72/), and miRTarBase (http://mirtarbase.cuhk.edu.cn/php/ index.php).

### Carbon-13 Urea Breath Test

None of the enrolled participants had a previous history of *H. pylori* eradication treatment. All patients were subjected to a carbon-13 (C13) urea breath test to detect* H. pylori* infection. Before the test, these patients had not received antibiotic treatment for at least 1 month and had avoided the use of proton pump inhibitors, potassium ion competitive acid blockers, or H2 receptor inhibitors for 2 weeks.

### Cell Culture, Transfection, Infection, and *Helicobacter pylori* Induction

Cell lines AGS and MKN-45 (Shanghai branch of the Chinese Academy of Sciences) were cultured in F12K and DMEM medium with 10% fetal bovine serum, respectively.

miR-196a/b-5p mimic and nonspecific mimic (NSM) were sourced from RiboBio in Guangzhou, China. The cells were transfected with 20 nanomoles of either the specific miR-196a/b-5p mimics or the NSM utilizing Invitrogen’s Lipofectamine RNAi MAX (Carlsbad, California).

The lentiviral expression plasmids for IGF2BP1 and miR-196a/b-5p were developed by Hanyin Biotechnology Limited in Shanghai, China. Cells were infected with 20 μL of IGF2BP1 lentivirus (2 × 10^8^TU/mL) either alone or in combination with 15 μL of miR-196a/b-5p lentivirus (1 × 10^9^TU/mL). A blank lentivirus (3 × 10^8^TU/mL) served as the control. The infected cells were utilized for subsequent in vitro or in vivo nude mouse experiments.

The *H. pylori* Sydney strain SS1 (Guangdong Microbial Culture Collection Center, China) was cultivated in a liquid nutrient solution, encompassed by an impermeable and airtight enclosure consisting of 85% nitrogen, 10% carbon dioxide, and a small fraction of 5% oxygen. AGS cells were seeded in 6-well plates (1 × 10^5^cells/plate) and induced by *H. pylori*.

### Protein Extraction and Western Blot

AGS cells transfected with either the miR196a/b-5p mimic or NSM were collected and lysed (Pierce; Thermo Fisher Scientific, Inc.). The western blot was performed according to the protocol. For western blot analysis, the primary antibodies were anti-human IGF2BP1 (no. ab184305, Abcam, USA), p-PI3K (no. ab138364, Abcam, USA), p-Akt (no. ab38449, Abcam, USA), or β-actin (no. db7283, diagbio, CHN) and the secondary antibody was horseradish peroxidase-conjugated goat anti-rabbit IgG (no. 7074, Sigma-Aldrich).

### Plasmid Construction and Luciferase Reporter Assay

The pmirGLO vector was employed to engineer recombinant plasmids harboring the native sequence (pmirGLO-h-IGF2BP1-WT) as well as the engineered variant of the 3’-untranslated regions (3’-UTR) of human IGF2BP1 cDNA (pmirGLO-h-IGF2BP1-MUT). Focusing on the 3’-UTR spanning nucleotides 1631-1637 of IGF2BP1 cDNA, this segment contains a putative miR-196a/b-5p interaction motif (ACTACCT) and was synthesizedviaPCR using genomic material procured from HEK293T cells. Subsequently, this fragment was cloned into the pmirGLO construct fused with the Renilla luciferase indicator. Point mutations were induced by modifying a sequence of seven nucleotides within the miR-196a/b-5p binding region, converting it to TGATGGA. This precise genetic engineering was executed using site-specific mutagenesis, employing the QuikChange XL kit (Stratagene, La Jolla, California).

The cells were seeded in 96-well plates (1.5 × 10^4^ 293T cells/plate); 24 hours later, the cells were transfected with either the native or mutant version of the IGF2BP1 3’ UTR as part of the pmirGLO luciferase reporting system. The cells were transfected with miR-196a/b-5p (100 nM) mimic or control at the same time using Invitrogen’s Lipofectamine™ 3000 system (product code L3000008). Then the luminescence signaling from luciferase activity was measured and quantified (Dual-Glo Luciferase Assay System, Promega). These operations were repeated in triplicates, and the overall assay series was replicated thrice.

### Transwell Cell Migration 5-Ethynyl-2’-deoxyuridine Apollo 488, and Colony Formation Assays

The AGS cells transfected with miR196a/b-5p mimic or NSM were placed into 24-well plates (2.5 × 10^4^cells/plate) with specialized inserts and Matrigel matrix (sourced from R&D Systems in the United States). Twenty-four hours later, the cells were stained with crystal violet (0.1%). Finally, the penetrating cells within 10 randomly chosen microscopic visual fields were quantified using inverted microscopy.

The AGS cells were seeded into 96-well assay plates (3 × 10^4^cells/plate). After 24 hours, the cells were transfected or infected with miR-196a/b-5p/NSM or miR-196a/b-5p/IGF2BP1 lentiviral vectors, respectively. Six hours later, the cells were stained with 5-ethynyl-2’-deoxyuridine (EdU) and DAPI, following the instructions of a RiboBio kit. Proliferating cells were examined using a fluorescence microscope, and the cell proliferation rate was determined using the following formula: (number of proliferating cells/total cells) × 100%.

The AGS cells were transfected or infected with miR-196a/b-5p/NSM or miR-196a/b-5p/IGF2BP1 lentivira. Twenty-four hours later, these cultures were subsequently seeded into 6-well plates (2 × 10^3^cells/well). Two weeks later, these cells were then preserved using a 4% paraformaldehyde solution and stained with crystal violet dye (0.1%). Colony quantifications were executed viaan optical microscope.

### Subcutaneous Tumor Xenograft Model

Female nude mice, aged between 6 and 8 weeks, were procured from Changzhou Kavins Experimental Animal Co., Ltd. The mice were housed in a sterile environment, with constant access to both nourishment and hydration. Seven days were allocated to permit them to adjust to new settings.

The MKN-45 cells were infected with either miR-196a/b-5p or a negative control lentivirus. The cell suspension (4 × 10^7^cells/100 microliters of saline solution) was administered subcutaneously to the mice. Assessments of tumor size were conducted employing the formula (volume = 0.5 × length × width^2^), with the initial measurement occurring on the 9th day following the inoculation. After 21 days, the mice were euthanized using 2% isoflurane anesthesia. 

### Statistical Analysis

The analytical procedures were enhanced by leveraging IBM SPSS Statistics 19.0 (IBM SPSS Corp.; Armonk, NY, USA). Continuous variables were presented as means along with their associated standard deviations (x- ± SD). The statistical difference in means among various categories was evaluated using a one-way Analysis of Variance (ANOVA). A *P*-value of less than .05 was considered an indicator of statistical relevance.

## Results

### The Expression Profiles of miRNAs Exhibited Notable Alterations During the Development of Gastric Mucosa Carcinogenesis

**Figure 1.**
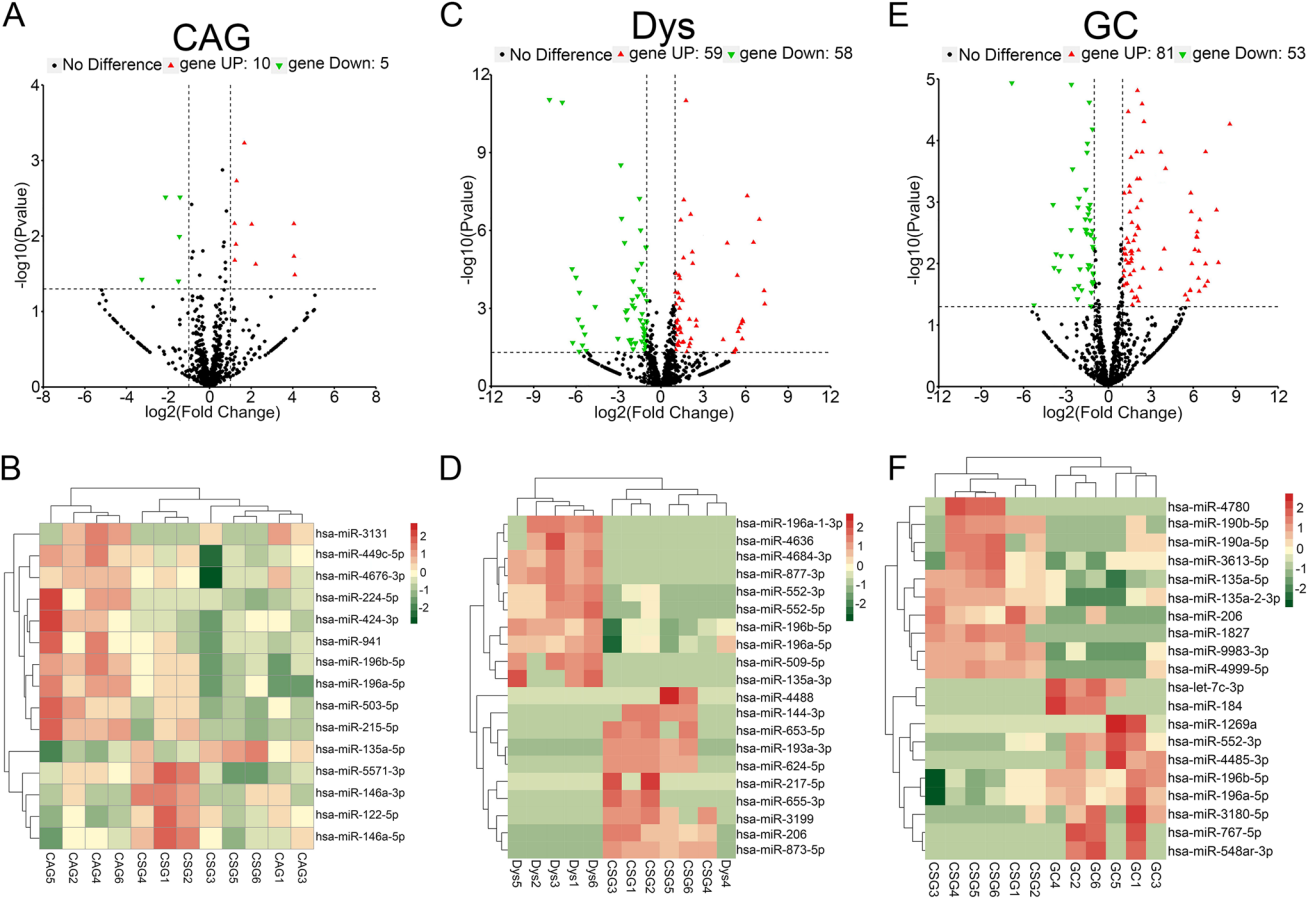
miRNA expression profiles in gastric mucosa with CSG, CAG, Dys, and GC. (A, C, E) A volcano plot of differentially expressed profiles of miRNAs. The red dots represent the number of upregulated miRNAs, and the green dots represent the number of downregulated miRNAs. The x-axis represents the multiple of difference, and the y-axis represents the *P* value. (B, D, F) A heat map of miRNA expression profile. The signal values in the figure range from −2 to +2. The red color represents high signal (upregulated), and the green color represents low signal (downregulated). The right column shows the names of significantly changed miRNAs. n = 6 for each group. CAG, chronic atrophic gastritis; CSG, chronic superficial gastritis; Dys, dysplasia; GC, gastric cancer.

The comparison with the control (CSG) group revealed that in the CAG cohort, a total of 15 miRNAs showed statistically deregulated (*P* < .05), 10 were increased, and 5 were decreased (Figure 1A). A heatmap represented these 15 dysregulated miRNAs (Figure 1B). In the Dys group, there were 117 miRNAs significantly affected (*P<*.05), with 59 upregulated and 58 downregulated (Figure 1C), and the top 20 impacted miRNAs were displayed in heatmap (Figure 1D). Finally, within the GC group, there were 134 deregulated miRNAs (*P<*.05), with 81 upregulated and 53 downregulated (Figure 1E), and the top 20 impacted miRNAs were displayed in heatmap (Figure 1F).

### *Helicobacter pylori* Infection Elevated the Expression Levels of miR-196a/b-5p, which Subsequently Modulated Tumor-Related Gene Ontology and Kyoto Encyclopedia of Genes and Genomes Pathways

**Figure 2.**
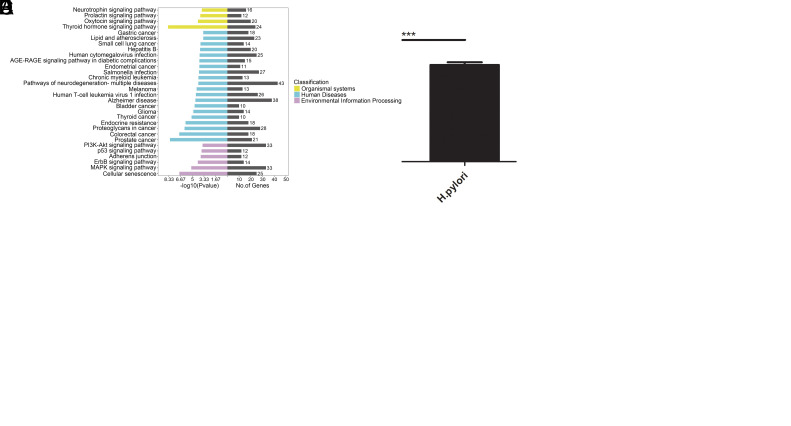
The continuously upregulated miRNAs and bioinformatic analysis. (A) The miRNAs continuously upregulated. The signal values in the figure range from −2 to 2. The red color represents a high signal (upregulated), and the green color represents a low signal (downregulated). The right column shows the names of differentially expressed miRNAs. (B) miR-196a-5p and (C) miR-196b-5p expression in AGS cells with *H. pylori* stimulation. AGS cells were stimulated with *H. pylori* for 12 hours, and the expression of miR-196a/b-5p was detected by RT-PCR. The x-axis represents different groups, PBS was used as the control, and the y-axis represents the relative expression of miR-196a-5p or miR-196b-5p. The study is repeated three times, *** *P* < .001. (D) The top 30 GOs that were identified to be regulated by the 5 upregulated miRNAs. The x-axis in the figure represents the proportion of differentially expressed genes enriched in the background GOs, the y-axis represents the gene function name. The length of the grey bar in the figure indicates the number of differentially expressed genes enriched; the longer the bars, the more GOs were regulated by the miRNAs. The colorful bars indicate the −log10 (*P* value) of the GOs, and the classification of the GOs is indicated by different colors. (E) The top 30 KEGGs that were identified as being regulated by the 5 upregulated miRNAs. The x-axis in the figure represents the proportion of differentially expressed genes enriched in the background pathways. The y-axis represents the name of the pathway. The length of the grey bar in the figure indicates the number of differentially expressed pathways enriched; the longer the bars, the more KEGGs were regulated by the miRNAs. The colorful bars indicate the −log10 (*P* value) of the KEGGs, and the classification of the KEGGs is indicated by different colors.

The analysis revealed that 5 miRNAs—miR-196a-5p, miR-196b-5p, miR-224-5p, miR-424-3p, and miR-941—were consistently upregulated in the CAG, Dys, and GC groups compared to the CSG group (Figure 2A). Given the crucial role of *H. pylori* in GC progression, its association with deregulated miRNAs was examined. Following *H. pylori* infection of AGS cells, miRNA expression was evaluated, revealing a significant increase in miR-196a/b-5p levels (Figure 2B and C). Bioinformatics analysis was conducted to identify the functions and pathways influenced by these 5 upregulated miRNAs in GC. Gene Ontology categories showed these miRNAs exerted regulatory effects on a wide spectrum of biological processes such as the regulation of activity in cyclin-dependent kinases involved with protein serine/threonine phosphorylation, the dynamics of cell growth and reproduction, adaptive physiological responses to varying levels of oxygen, and also the process of cellular breakdown (Figure 2D). Simultaneously, the KEGG analysis showed these miRNAs regulated the pathways pertinent to the advancement of GC, including PI3K-Akt, p53, and MAPK signaling network (Figure 2E).

### The Expression of miR-196a/b-5p in Gastric Precancerous Lesions of Patients with and Without *Helicobacter pylori* Infection

**Figure 3.**
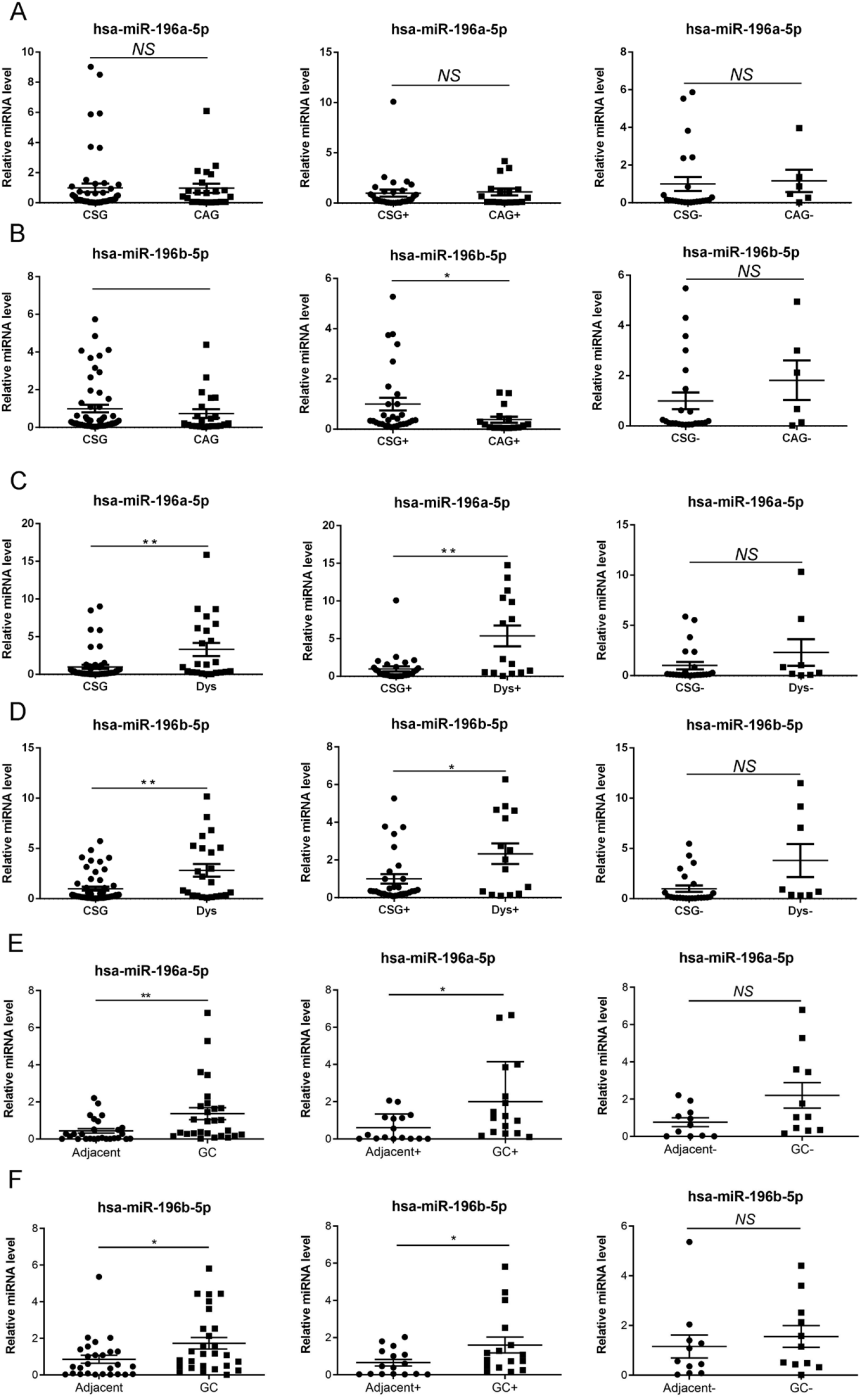
Verification of miR-196a/b-5p expression in the gastric mucosa tissues with CSG, CAG, Dys, and GC. (A-F) RT-PCR verified the miR-196a/b-5p expression in CSG (n = 85), CAG (n = 26), Dys (n = 32), and GC(n = 27) groups. Tissue samples from patients positive for *H. pylori* are shown as CSG+, CAG+, Dys+, GC+, and Adjacent+; mucosa samples from patients negative for H. pylori are shown as CSG-, CAG-, Dys-, GC-, and Adjacent-. The x-axis represents different groups, and the y-axis represents the relative expression of miR-196a-5p or miR-196b-5p, * *P* < .05, ** *P* < .01.

To further analyze the expression of miR-196a/b-5p in gastric precancerous lesions, a larger sample size was examined using the RT-qPCR technique. The results indicated that miR-196a/b-5p levels were significantly elevated in Dys or GC tissues compared to CSG or adjacent tissues. Upon stratifying patients into *H. pylori*-positive and *H. pylori*-negative groups, a significant upregulation was observed in *H. pylori*-positive individuals (*P<*.01), while no notable difference was found in *H. pylori*-negative individuals (*P>*0.01) (Figures 3C-F). Upon meticulous analysis, there appeared to be no noteworthy variances between CAG and CSG (Figures 3A and B).

### miR-196a/b-5p Facilitated the Progression of Gastric Cancer Both In Vitro and In Vivo

**Figure 4.**
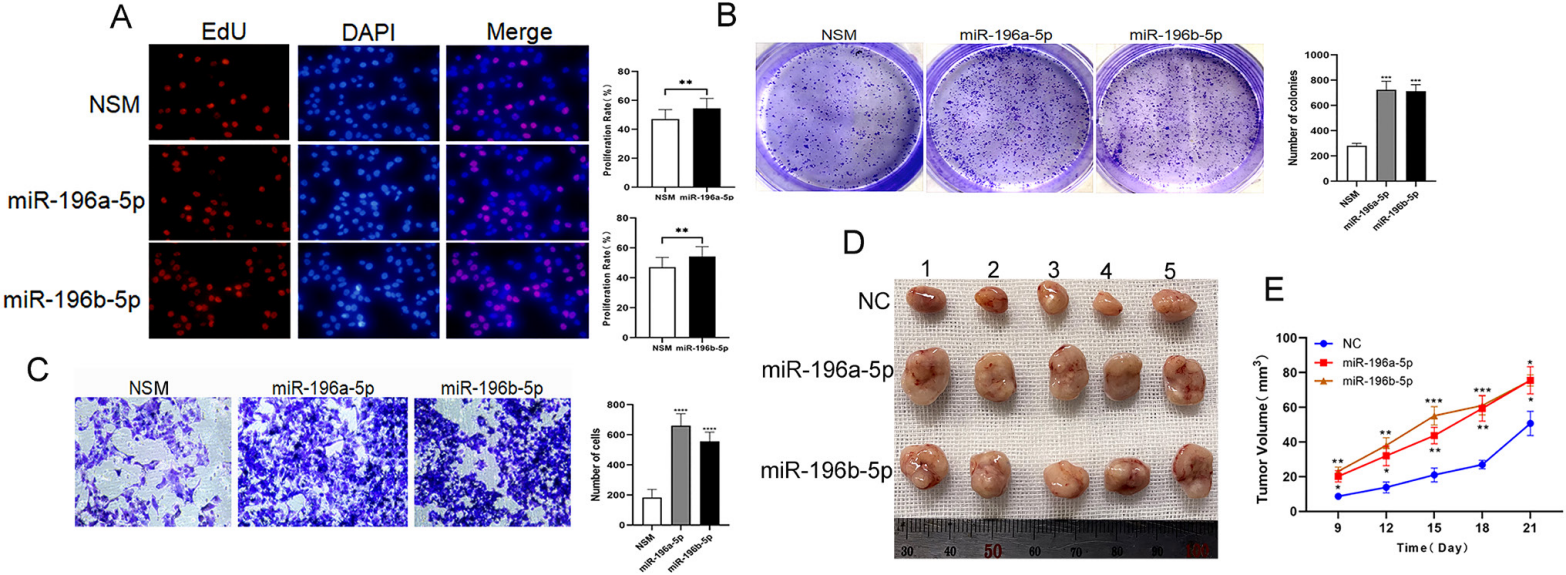
Promoting roles of miR-196a/b-5p in GC cell proliferation and migration were studied in vitro and in vivo. The AGS cells transfected with miR-196a/b-5p mimic or NSM for 48 hours. (A) Proliferation study with EdU assay. A total of 3 × 10^3^ transfected AGS cells per well in 96-well plates were cultured for 24 hours, EdU-positive cells are stained with red, and nuclei are stained with DAPI (blue). The percentage of EdU-positive cells (proliferation rate%) in 10 randomly selected microscopic fields are counted. Mean (±SEM) values of 3 independent experiments are presented. **P < .01. (B) Cell cloning study. A total of 1 × 10^3^ well transfected cells seeded into 6-well plates and cultured; after 14 days, the cells are fixed and stained. Cell clones are stained with purple by 0.1% crystal violet; the number of clones in 10 randomly selected fields is then counted under an optical microscope. Data are shown as the mean value of 3 independent experiments. Bars represent the mean (±SEM) number of colonies with more than 50 cells. ***P < .001. (C) Migration study with transwell: 2.5 × 10^4^ /well transfected cells were cultured in 24-well plates with Matrigel-coated membranes. Cells in the bottom chamber are fixed and stained purple by 0.1% crystal violet, and the number of stained cells in 10 randomly selected fields is counted. Data are shown as the mean value of 3 independent experiments. Bars represent the mean (±SEM) number of invaded cells per field. ***P < .001. (D) MKN-45 cells derived from the subcutaneous GC tumor of the mouse model are used; 21 days after modeling, the mice are euthanized and death was confirmed by the cervical dislocation method. The tumors are removed (n = 5 for each group). (E) The tumor volume is recorded and shown by line chart. The x-axis represents different time points, and the y-axis represents the tumor volume (mm^3^),*P < .05, **P < .01, ***P <.001.

The gathered evidence presents a compelling case for miR-196a/b-5p’s pivotal role in the malignant transformation of gastric epithelial cells. Then the EdU, colony formation, and transwell assays were performed. Data procured from EdU labeling and colony formation analyses indicated a marked enhancement in GC cell replication following the overexpression of miR-196a/b-5p (Figures 4A and B). Moreover, transwell analysis outcomes attested to the augmented migratory proficiency of GC cells consequent to miR-196a/b-5p upregulation (Figure 4C). Further, the in vivonude mice experiment showed elevated levels of miR-196a/b-5p substantially accelerated tumor development (Figures 4D and E). The results collectively underscore the importance of targeting miR-196a/b-5p within therapeutic strategies aimed at mitigating GC pathogenesis.

### miR-196a/b-5p was Found to Target IGF2BP1, Leading to a Reduction in its Expression

**Figure 5.**
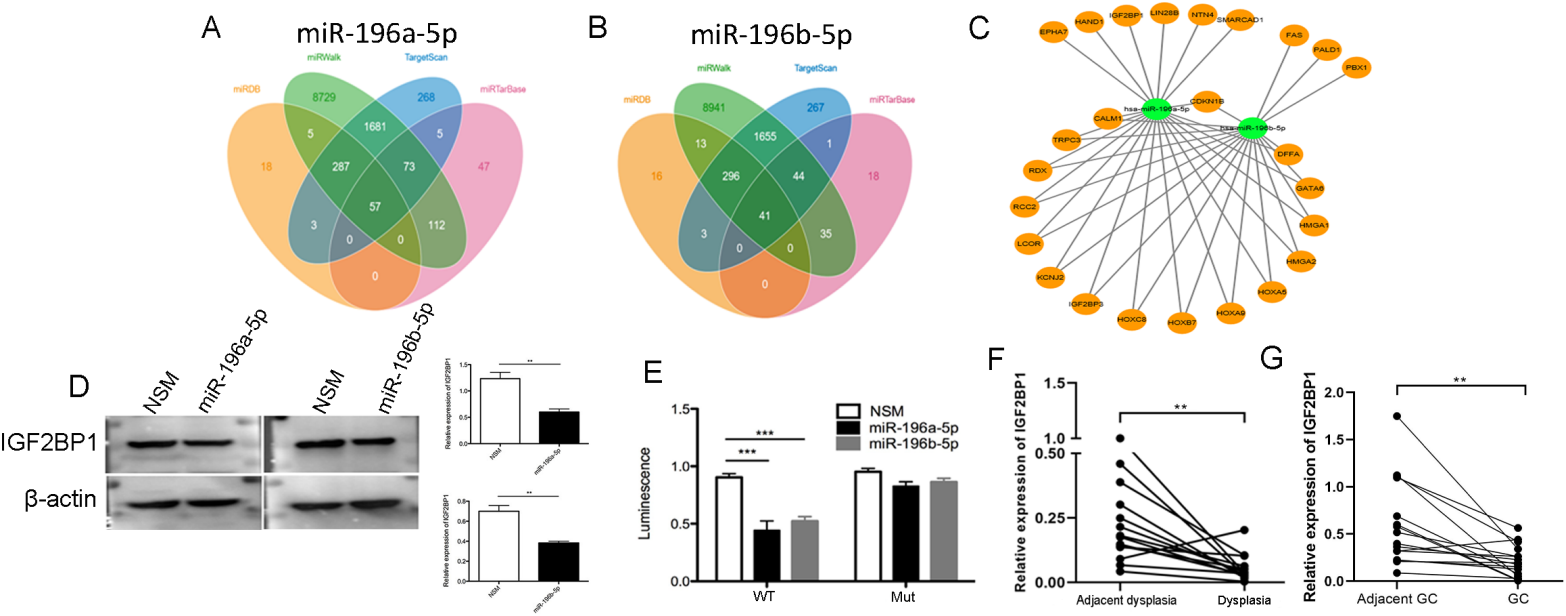
Targeted genes were verified by western blot and luciferase reporter assay. (A, B) The idiograms of targeted genes of miR-196a/b-5p forecasted from miRBD, miRWalk, TargetScan, and miRTarBase databases, the number of cross-expressed proteins in the four databases is shown. (C) The 25 cross-expressed proteins which were related to tumorigenesis are shown according to the data above. (D) Western blot assay is performed to detect the expression of IGF2BP1 in AGS cells of miR-196a/b-5p overexpression (miR-196a/b-5p) and control (NSM). β-actin was used as the internal control for total proteins, and the quantification of western blot is shown in the bar chart. Data are shown as the mean value of 3 independent experiments ± SEM, **P < .01. (E) Luciferase reporter assay. 293T cells were grown and co-transfected with miR-196a/b-5p mimic (miR-196a/b-5p) or non-specific mimic (NSM) plus wild type of the IGF2BP1 3’-UTR (WT) or the mutant 3’-UTR (Mut) and then subjected to the Renilla luciferase activity assay. The mutational sites (1631-1637) were listed and marked in red. Data represent mean value of three independent experiments ± S.E.M. *P < .05, **P < .01. (F, G) IGF2BP1 mRNA expression was detected using RT-qPCR. The x-axis represents 15 dysplasia or 16 GC and matched adjacent tissues from the patients who were *H. pylori* positive, the y-axis represents the relative IGF2BP1 mRNA expression. Bars represented the mean (±SEM) values of 3 independent experiments. **P < .01.

Bioinformatics methods predicted that miR-196a-5p targets a total of 57 genes, while miR-196b-5p targets 41 genes (Figure 5A and B). Among these, 25 overlapping genes associated with tumor progression (Figure 5C). The IGF2BP1 was the sole gene inhibited by both miR-196a-5p and miR-196b-5p, confirmed by western blot analysis (Figure 5D) and luciferase reporter assay (Figure 5E) separately. Furthermore, RT-qPCR was employed to assess IGF2BP1 mRNA levels in gastric mucosa with dysplasia or GC, demonstrating that IGF2BP1 mRNA was significantly downregulated in dysplastic or cancer tissues compared to adjacent normal tissues (Figures 5F and G).

### miR-196a/b-5p has the Ability to Partially Counteract the Anticarcinogenic Effects of IGF2BP1 in Gastric Cancer Cells.

**Figure 6.**
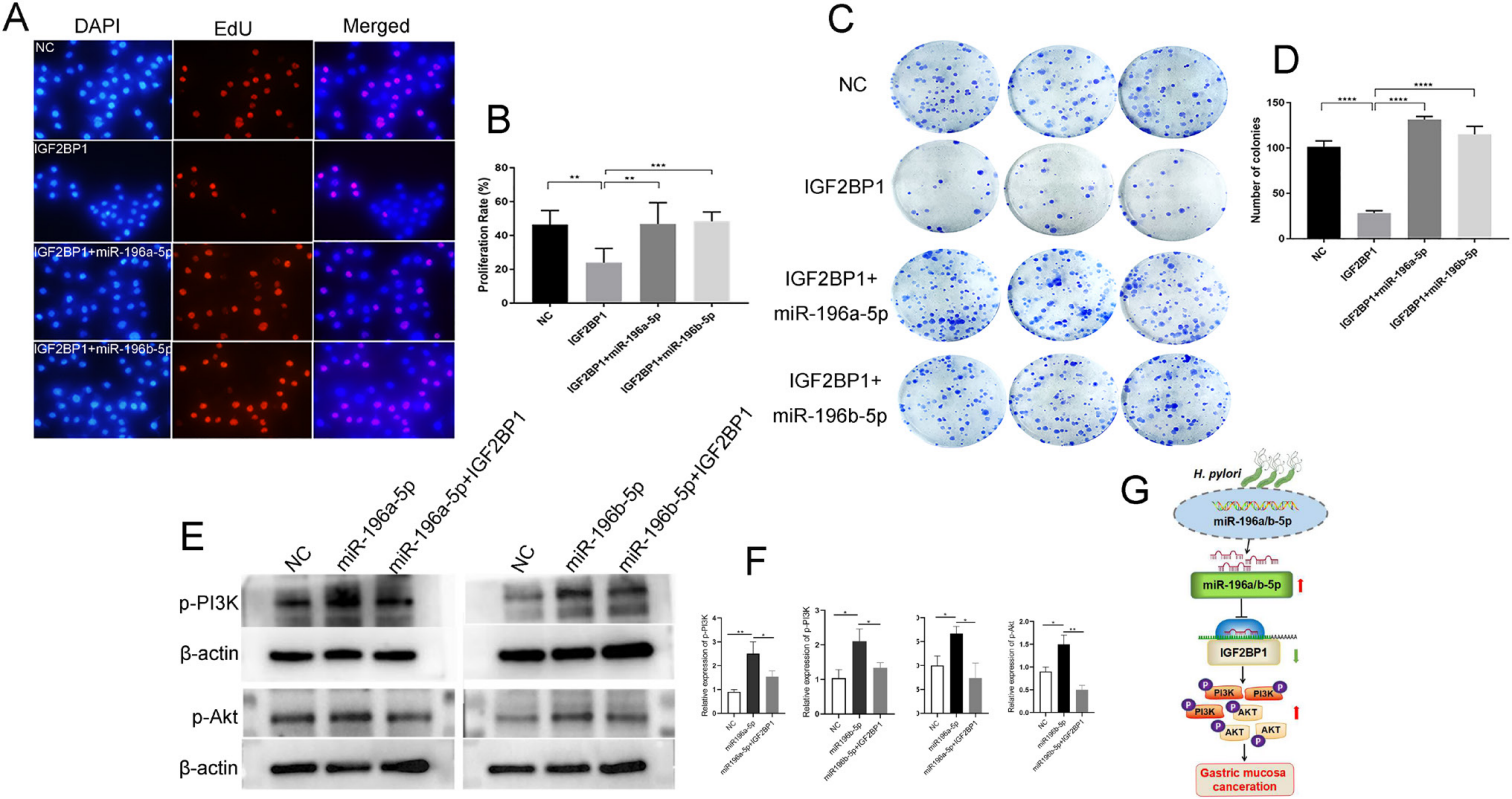
Targeted effect of miR-196a/b-5p on IGF2BP1 in GC. The AGS cells were infected with IGF2BP1, IGF2BP+miR-196a-5p, IGF2BP+miR-196b-5p, or negative control (NC) lentivirus for 48 hours. (A) Proliferation study with EdU assay. A total of 3 × 10^3^ infected AGS cells per well in 96-well plates are cultured for 24 hours, EdU-positive cells were stained red, and nuclei were stained with DAPI (blue). (B) The percentage of EdU-positive cells (proliferation rate%) in 10 randomly selected microscopic fields is counted. Mean (±SEM) values of three independent experiments are presented. **P < .01, ***P < .001. (C) Cell cloning study. A total of 1 × 10^3^/well infected cells were seeded into 6-well plates and cultured; after 14 days, the cells were fixed and stained, cell clones were stained purple using 0.1% crystal violet. (D) The number of clones in 10 randomly selected fields was counted under an optical microscope, as represented via a histogram. Data are shown as the mean value of 3 independent experiments. Bars represent the mean (±SEM) number of colonies with more than 50 cells. ***P < .001. (E, F) The proteins in the PI3K-Akt signaling pathway were detected by Western blot analysis. The proteins in the AGS cells infected with IGF2BP1, IGF2BP+miR-196a-5p, IGF2BP+miR-196b-5p, or negative control (NC) lentivirus above were detected. β-actin was used as the internal control for total proteins, and the quantification of western blot is shown in the bar chart. Data are shown as the mean value of three independent experiments±SEM, **P < 0.01. (G) The pattern diagram of *H. pylori*/miR-196a/b-5p roles in gastric mucosa canceration. The red arrow indicates upregulation, and the black arrow indicates downregulation. Targeting is indicated by a suppression symbol.

The role of IGF2BP1 in promoting GC cell proliferation was assessed through EdU incorporation and cell cloning assays. The study noted a decrease in the growth rate of gastric carcinoma cells when there was an upsurge in the concentration of IGF2BP1. However, when cells were co-transfected with miR-196a-5p or miR-196b-5p, this inhibition was partially reversed (Figures 6A-D). The upregulation of miR-196a/b-5p increased the levels of phosphorylated PI3K and Akt proteins, but this increase was partially mitigated by IGF2BP1(Figures 6E and F). The implication here is that miR-196a/b-5p could potentially promote the development of GC through its interaction with IGF2BP1 and subsequent activation of the PI3K-Akt signaling cascade (Figure 6G).

## Discussion

*Helicobacter pylori* is identified as the primary risk factor for GC, responsible for nearly 90% of new non-cardia GC cases.^9^ Numerous studies indicate that *H. pylori* may facilitate the progression of GC through miRNA pathways.^10^ One particular study found that miR-222-3p was highly expressed in human GC tissues infected with *H. pylori* and facilitated the advancement of GC by targeting HIPK2.^11^ Another investigation revealed a significant downregulation of miR-101 in cases of *H. pylori*-related GC and that high miR-101 expression inhibits the growth of GC cells.^12^ A recent investigation into the miRNA expression profiles in early and advanced GC did not identify any miRNAs associated with inflammation and atrophy.^13^ In this study, miRNA expression profiles in the gastric mucosa from inflammation to cancer was analyzed, observing that the expression of miRNAs altered significantly through the stages of CSG, CAG, Dys, and GC, with several of these miRNAs previously reported to be involved in GC.^14^

miR-196a has been reported to be upregulated in a diverse array of cancer types, positioning its abundance in tumor specimens and circulatory fluids as a potentially invaluable biomarker for the prognosis and diagnosis of these diseases.^15^ Furthermore, empirical evidence suggests a linkage between heightened expression of miR-196a-5p and both the propensity for lymphatic dissemination and progression through clinical phases, implicating its significant influence on the pathogenesis of GC.^16^ miR-196b-5p has emerged as a promising candidate for use as an oncogenic miRNA biomarker. Its presence may predict the effectiveness of chemotherapy in individuals battling GC.^17^ This molecular marker has been associated with increased growth and infiltrative characteristics of GC cells, suggesting its pivotal role in the advancement of this malignancy.^18^^,^^19^ However, the expression levels of miR-196a/b-5p in precancerous gastric mucosal lesions and the mechanisms associated with cancer development remain unexplored. In this study, microarray analysis revealed that miR-196a/b-5p expression is elevated in the gastric mucosa affected by CAG, Dys, and GC compared to those with CSG, suggesting that miR-196a/b-5p may play a significant role in the development of precancerous and cancerous gastric lesions. Additionally, miR-196a/b-5p was found to be elevated in gastric tissues with Dys or GC, especially in tissues from *H. pylori*-positive individuals. This implies that *H. pylori* is implicated in modulating the levels of miR-196a/b-5p, which is essential in the progression of both pre-malignant and malignant growths within the gastric epithelium.

In pursuit of a deeper understanding of miR-196a/b-5p involvement in GC, the research comprised experimental work both in cellular models and within living organisms. The evidence gathered suggests a promoting effect of miR-196a/b-5p on both cell proliferation and migratory capabilities in GC, alongside an expedited growth rate of subcutaneous GC tumors in a nude mouse model. miRNAs typically modulate gene expression post-transcription by binding to messenger RNA. Furthermore, IGF2BP1 was predicted and confirmed to be the solitary gene demonstrably repressed by both miR-196a-5p and miR-196b-5p and solidified the implication of IGF2BP1 downregulation in the mechanistic action of miR-196a/b-5p in GC.

IGF2BP1, recognized as a modulator of m6A methylation, has been implicated as either an oncogenic factor or a tumor suppressor.^20^ In this study, it was found that IGF2BP1 mRNA was decreased in dysplastic tissues. Overexpression of IGF2BP1 led to a reduced proliferation rate in GC cells. This inhibition could be partially reversed by miR-196a/b-5p, which activates the PI3K/Akt signaling pathway. However, IGF2BP1 can partially attenuate this effect. Prior research has confirmed that IGF2BP1 can engage c-Myc mRNA, augmenting its oncomodulatory activity, which fuels the pathogenesis of GC.^21^ Patients with GC exhibiting elevated levels of IGF2BP1 mRNA showed significantly worse overall survival.^22^ These findings contrast with those of this study, potentially due to variations in the cell lines and study populations examined. Further research is necessary to confirm the role of IGF2BP1 in GC.

This research has several limitations. The findings indicated that *H. pylori* infection influenced the expression of miR-196a/b-5p, which subsequently targeted the m(6)A methylation modification regulator IGF2BP1, thereby promoting GC tumorigenesis. However, the specific signaling pathway involved remains unclear and necessitates further investigation. The significant expression of miR-196a/b-5p in precancerous lesions suggests its potential regulatory roles. Future studies would benefit from employing more reliable research models, such as organoids, to confirm these effects. In addition, this study defines *H. pylori* infection status based solely on a single method of C13 urea breath test. To more accurately reflect the *H. pylori* infection status, other methods such as serological antibody detection, rapid urease test on biopsy tissue during gastroscopy, and* H. pylori* antigen detection in stool will be combined one or more as diagnostic criteria.

In conclusion, the regulatory mechanism involving *H. pylori*, miR-196a/b-5p, and IGF2BP1 in GC was elucidated through bothin vitro andin vivoapproaches. The findings shed light on the underlying causes of GC connected to *H. pylori* infection and present novel viewpoints for the preliminary identification and management of precancerous conditions in the stomach as well as GC itself.

## Data Availability

The data used to support the findings of this study are available from the corresponding author upon reasonable request.
